# BMP4-Induced Suppression of Breast Cancer Metastasis Is Associated with Inhibition of Cholesterol Biosynthesis

**DOI:** 10.3390/ijms25179160

**Published:** 2024-08-23

**Authors:** Lap Hing Chi, Andrew D. Redfern, Terry C. C. Lim Kam Sian, Ian P. Street, Allan D. Burrows, Suraya Roslan, Roger J. Daly, Robin L. Anderson

**Affiliations:** 1 Olivia Newton-John Cancer Research Institute, Heidelberg, VIC 3084, Australia; leo.chi@onjcri.org.au (L.H.C.);; 2 School of Cancer Medicine, La Trobe University, Bundoora, VIC 3083, Australia; 3 Medical School, University of Western Australia, Perth, WA 6009, Australia; andrew.redfern@health.wa.gov.au; 4 Cancer Program, Monash Biomedicine Discovery Institute, Clayton, VIC 3168, Australia; terry.lim@monash.edu (T.C.C.L.K.S.); roger.daly@monash.edu (R.J.D.); 5 Department of Biochemistry & Molecular Biology, Monash University, Clayton, VIC 3168, Australia; 6 Children’s Cancer Institute, University of New South Wales, Sydney, NSW 2052, Australia; istreet@ccia.org.au; 7 Department of Surgery, St. Vincent’s Hospital, Fitzroy, VIC 3065, Australia; suraya.roslan@svha.org.au; 8 Department of Clinical Pathology, The University of Melbourne, Parkville, VIC 3052, Australia

**Keywords:** breast cancer, metastasis, BMP4, cholesterol biosynthesis, statins

## Abstract

We reported previously that in preclinical models, BMP4 is a potent inhibitor of breast cancer metastasis and that high BMP4 protein levels predict favourable patient outcomes. Here, we analysed a breast cancer xenograft with or without enforced expression of BMP4 to gain insight into the mechanisms by which BMP4 suppresses metastasis. Transcriptomic analysis of cancer cells recovered from primary tumours and phosphoproteomic analyses of cancer cells exposed to recombinant BMP4 revealed that BMP4 inhibits cholesterol biosynthesis, with many genes in this biosynthetic pathway being downregulated by BMP4. The treatment of mice bearing low-BMP4 xenografts with a cholesterol-lowering statin partially mimicked the anti-metastatic activity of BMP4. Analysis of a cohort of primary breast cancers revealed a reduced relapse rate for patients on statin therapy if their tumours exhibited low BMP4 levels. These findings indicate that BMP4 may represent a predictive biomarker for the benefit of additional statin therapy in breast cancer patients.

## 1. Introduction

Breast cancer is a leading cause of cancer-related death in women, with the majority of deaths being due to uncontrolled metastatic disease. The 5-year survival rate for patients diagnosed with metastatic breast cancer is still only 29% [1]. In de novo metastatic disease, where primary and metastatic disease are both present, the progression of metastatic disease is relatively common, despite effective control of the primary tumour, indicating a biological disconnect between proliferative biologies. This also likely underlies, at least in part, the finding that surgical removal of the primary tumour does not improve quality of life or survival when distant metastases are also present [2].

We and others have shown that bone morphogenetic protein 4 (BMP4) is a potent inhibitor of breast cancer metastasis [3,4,5,6,7], although other studies report contradictory findings for the role of BMP4 [8,9]. We also noted that high BMP4 exerted a selective suppressive effect on metastatic disease without impacting behaviour of the primary tumour, suggesting that the anti-metastatic pathways governed by BMP4 might have substantial relevance to metastatic cancer control in the clinic.

BMP4 is a cytokine in the BMP/TGFβ family and is well recognised as a regulator of development and maintenance of homeostasis, signalling through both canonical and non-canonical pathways, depending on the context [10,11]. Canonical signalling engages SMAD1/5/9 and SMAD4 to drive the transcription of genes that can regulate cancer growth and metastasis. BMP4 can also signal through a non-canonical pathway, particularly in cells with loss of SMAD4, a common tumour suppressor gene, resulting in the activation of PI3K/AKT, NF-κB and MAPK pathways capable of promoting tumour progression [12]. However, the mechanisms by which BMP4 regulates metastasis are still not fully elucidated. At the cellular level, BMP4 reduces the number and activity of myeloid-derived suppressor cells which have an immunosuppressive role in cancer [3]. However, more recently, we have shown that BMP4 is also able to suppress metastasis in immune-compromised mice, indicating a second mechanism unrelated to immune responses [4].

In this study, we set out to identify further mechanisms underlying the protective effect of BMP4, with the goal of translating our findings for patient benefit. We re-analysed the transcriptomic data from tumour cells recovered from orthotopic primary breast MDA-MB-231HM tumours in immunocompromised mice with or without enforced expression of BMP4 [5] and we also completed a proteomic analysis of tumour cell responses to BMP4 exposure. Cholesterol biosynthesis was identified as a core pathway that was significantly suppressed by active BMP4 signalling. We further explored the relationship and interactions between BMP4, cholesterol biosynthesis and statin usage in our preclinical model and in patients, noting that cholesterol has been implicated in breast cancer progression and that cholesterol-lowering statin drugs have been associated with primary and secondary cancer protection.

## 2. Results

### 2.1. Alteration in the Transcriptome by BMP4

To identify therapeutically targetable pathways regulated by BMP4 to suppress metastatic disease, we interrogated a previously generated RNA sequencing dataset derived from breast cancer cells isolated from primary xenograft tumours [5]. The human breast cancer line, MDA-MB-231HM (231-HM), metastasises spontaneously from the primary mammary tumour to the lungs, liver and bone in immunocompromised NSG mice. Following enforced expression of BMP4 in these cells, metastasis to these organs is profoundly inhibited, whilst primary tumour growth is not altered [5]. Recovery of the tumour cells from the primary tumours by enzymatic disaggregation and flow cytometry based on their expression of TurboGFP allowed for selective analysis of the influence of BMP4 on gene expression in vivo. RNA sequencing revealed genes whose expression was altered by the presence of BMP4 in the tumour cells [5]. Here, we have utilised this dataset to identify genes and pathways altered by BMP4 expression in vivo.

**Figure 1:**
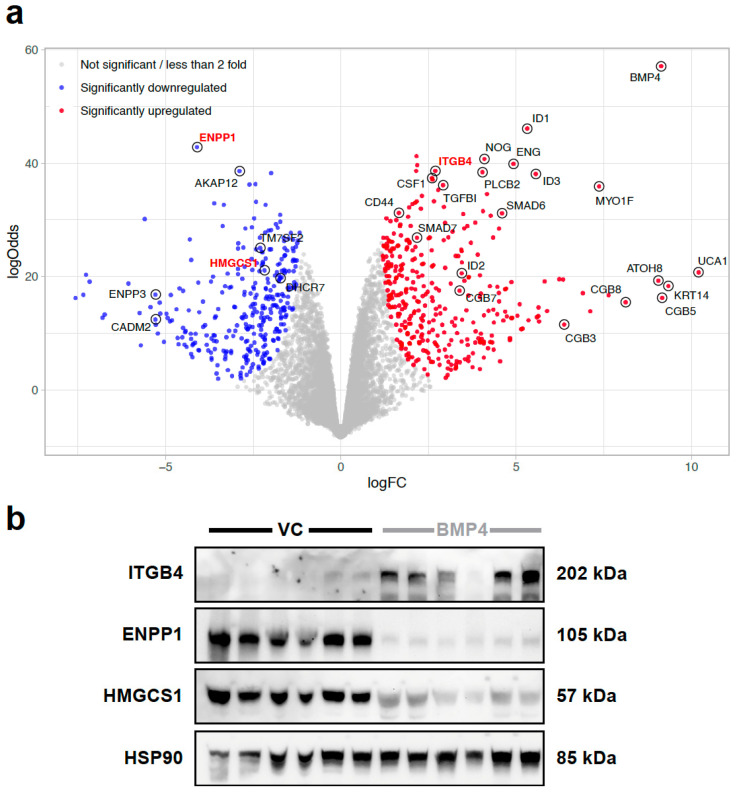
RNA sequencing analysis reveals BMP4 target genes that may regulate breast cancer metastasis. (**a**) Volcano plots highlighting significantly up- and downregulated genes by BMP4 in 231-HM tumours. Tumour cells were recovered from primary tumours following disaggregation and flow cytometry. Statistical analysis was completed using limma 3.42.2 package in R. (**b**) Western blotting validation of changes in protein levels of BMP4 target genes that were identified in RNA sequencing analysis (annotated in red in panel (**a**)). Proteins were extracted from resected 231-HM primary tumours with modified expression of BMP4. HSP90 was used as loading control. VC, vector control.

A volcano plot highlighting the genes most consistently and strongly regulated by BMP4 in 231-HM tumours is presented in Figure 1a. This revealed a number of known BMP4 target genes (*ID1*, *ID3* and *SMAD6*) and others that were identified in a recent publication [13], including urothelial cancer-associated 1, *UCA1*, and atonal BHLH transcription factor 8, *ATOH8*, as being amongst the most significantly upregulated genes by BMP4 (Figure 1a), confirming in 231-HM tumours the known transcriptomic regulation by BMP4.

Other novel BMP4-regulated genes emerged from this analysis, including Ectonucleotide pyrophosphatase/phosphodiesterase 1 (*ENPP1*), a known suppressor of anti-tumour STING signalling [14], showing potent downregulation by BMP4. Of note, *ENPP3* was also downregulated by BMP4 (Figure 1a). In contrast, myosin 1f (*MYO1F*), a gene that is known to mediate the polarisation and migration of immune cells [15,16] and which we have already shown to be a metastasis suppressor [5], was upregulated by BMP4. Other genes also upregulated by BMP4 that contribute to the formation of hemidesmosomes [17], including integrin β4 (ITGB4) and keratin 14 (*KRT14*), and a number of chorionic gonadotropin β genes (*CGB3/5/7/8*) encoding subunits of a hormone that has been implicated in breast cancer progression [18], were also noted. Of particular relevance here was the observation of the downregulation by BMP4 of HMGCS1, required for the synthesis of mevalonate, a precursor of cholesterol [19]. We confirmed the observed transcriptional upregulation of ITGB4 and the downregulation of ENPP1 and HMGCS1 in BMP4-expressing primary tumours at the protein level (Figure 1b).

### 2.2. Alteration in the Proteome by BMP4

**Figure 2:**
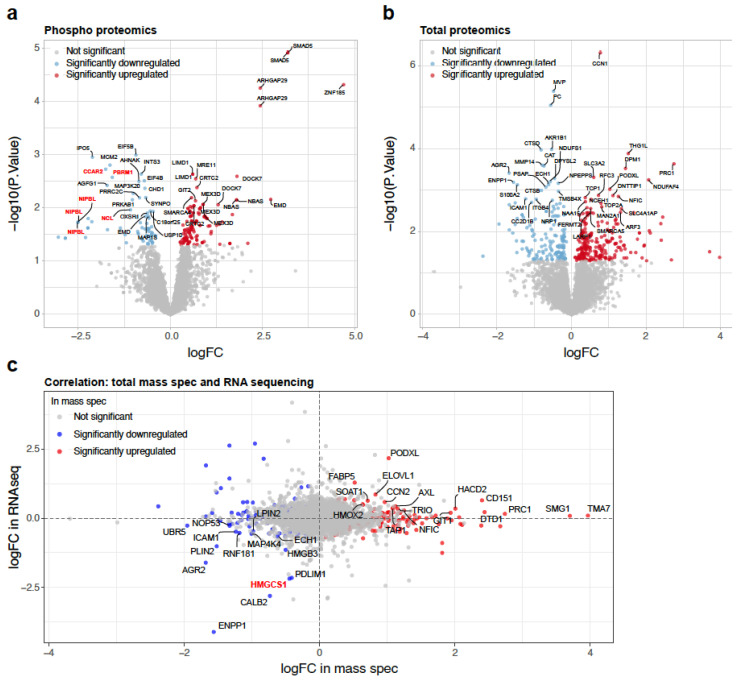
Mass spectrometric analysis of 231-HM tumour cells treated with recombinant human BMP4 in vitro. (**a**) Volcano plot highlighting significantly up- and downregulated phosphopeptides following 45 min of exposure to recombinant BMP4 (20 ng/mL). Proteins that are implicated in cholesterol biosynthesis are annotated in red. (**b**) Volcano plot highlighting significantly up- and downregulated total proteins following 24 h of exposure to recombinant BMP4 (40 ng/mL). (**c**) Comparison of BMP4-induced transcriptomic changes in vivo (Y axis) to BMP4-induced total proteomic changes (X axis) in vitro. Statistical analysis in (**a**,**b**) was completed using limma package in R.

To further explore BMP4-mediated mechanisms of metastasis inhibition, we undertook a mass spectrometry-based characterisation of the phosphoproteome and the total proteome of 231-HM cells following 45 min or 24 h of recombinant BMP4 treatment, respectively. To assess changes in phosphoproteins, we exposed the cells briefly to a lower concentration of BMP4. For changes in the total proteome, we exposed the cells for 24 h, using a higher concentration of BMP4 to maintain its presence over the extended timeframe. A volcano plot highlighting a number of significantly upregulated and downregulated phosphorylated peptides following 45 min of exposure to recombinant BMP4 is presented in Figure 2a. As anticipated, a 9.1-fold increase in phosphorylated SMAD5 (on either S463 or S465) was observed. Notably, several proteins that were less phosphorylated after recombinant BMP4 treatment have been implicated in the regulation of cholesterol biosynthesis, including cell cycle and apoptosis regulator 2 (CCAR2 on S671) [20], NIPBL cohesion loading factor (NIPBL on S1089, S1090 and S1096) [21], nucleolin (NCL on S206) [22] and polybromo 1 (PBRM1 on S13) [23] (Figure 2a).

Analysis of the total proteome highlighted proteins that were significantly upregulated and downregulated after 24 h of recombinant BMP4 treatment in 231-HM cells (Figure 2b). The correlation between BMP4-induced protein expression changes in the mass spectrometric analysis and transcript expression changes in the RNA sequencing analysis is presented as an XY plot in Figure 2c. ENPP1 and anterior gradient protein 2 homolog (AGR2) were consistently downregulated by BMP4 in vitro and in vivo at the transcript and protein level (Figure 2c). Similar to ENPP1 [14], AGR2 has been reported to have tumour-promoting activity, including the promotion of proliferation, migration, invasion and therapy resistance [24,25,26,27,28]. Importantly, HMGCS1 was also consistently downregulated by BMP4 (Figure 2c).

### 2.3. BMP4 Suppresses Cholesterol Biosynthesis

**Figure 3:**
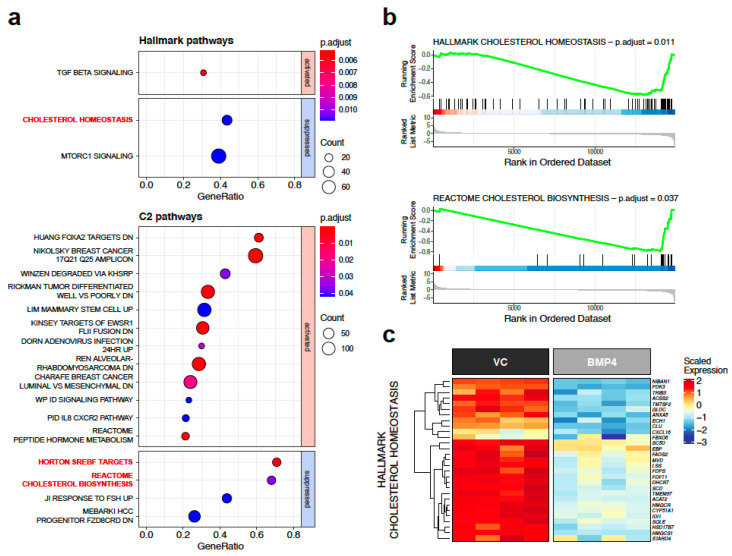
BMP4 negatively regulates cholesterol biosynthesis genes in 231-HM xenograft tumours. (**a**) Dot plots highlighting most significantly regulated Hallmark and C2 pathways in BMP4-expressing 231-HM xenograft tumours. Pathways that relate to cholesterol biosynthesis are annotated in red. Statistical analysis was completed using clusterProfiler 4.8.3 package in R. (**b**) Enrichment plots of BMP4-induced gene expression changes that relate to Hallmark cholesterol homeostasis pathway and Reactome cholesterol biosynthesis pathway. Data visualisation was completed using enrichplot 1.20.3 package in R. (**c**) Heatmap of BMP4-induced gene expression changes in Hallmark cholesterol homeostasis pathway. Data visualisation was completed using ComplexHeatmap 2.16.0 package in R.

Gene set enrichment analysis (GSEA) of the transcript data was completed using the MSigDB Hallmark and C2 datasets [29,30] to identify signalling pathways that were significantly modulated by BMP4 (Figure 3). Three of the most significantly suppressed pathways were all associated with the biosynthesis of cholesterol (Figure 3a). As visualised through barcode plots in Figure 3b, the majority of genes in the cholesterol homeostasis pathway (Hallmark gene set) and in the cholesterol biosynthesis pathway (Reactome gene set) were markedly downregulated by BMP4 in vivo.

Importantly, the expression of BMP4 did not affect the growth of primary tumours and the RNA sequencing data were generated from MDA-MB-231HM cells resected at the same time point [5]. Therefore, the changes observed in BMP4-expressing tumours were not driven by factors such as the rate of tumour growth. Given the profound inhibition of individual cholesterol homeostasis genes by BMP4 (Figure 3c) and the increasingly recognised pro-tumourigenic roles of this pathway in breast cancer progression [31,32,33], we explored further the interactions between BMP4 signalling and cholesterol biosynthesis and the feasibility of therapeutically targeting this pathway to reproduce the anti-metastatic impact of BMP4.

**Figure 4:**
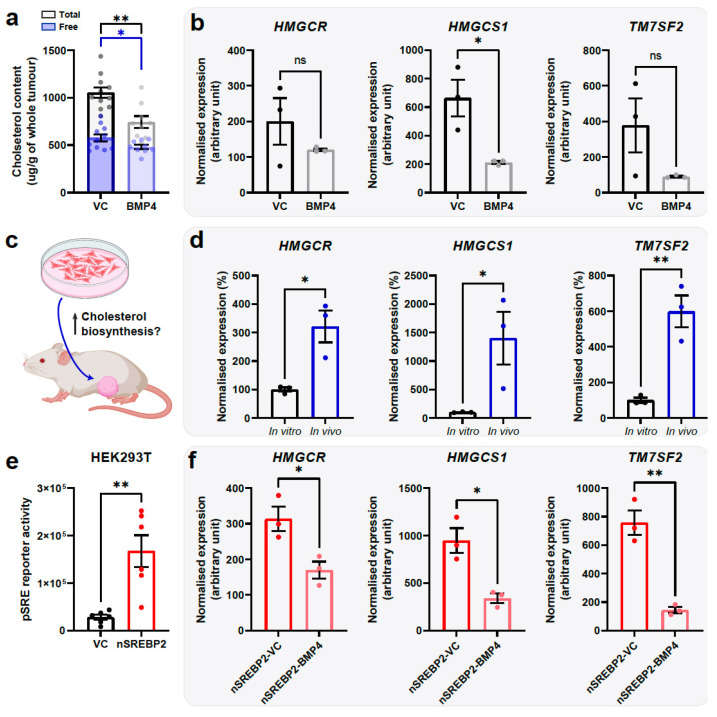
Validation of suppression of cholesterol biosynthesis by BMP4 in vivo and in vitro. (**a**) Quantitation of free (annotated in blue) and total (annotated in grey) cholesterol levels in 231-HM primary tumours. n ≥ 9/group, mean ± SEM. (**b**) RT-qPCR analysis of expression of cholesterol biosynthesis genes in 231-HM cells cultured in 0.5% serum. n = 3/group, mean ± SEM. (**c**,**d**) Expression of genes that relate to cholesterol biosynthesis both in vitro and from primary tumours in vivo. In (**d**), RNA was extracted either from in vitro cultured 231-HM cells or from resected 231-HM xenograft tumours. n = 3/group, mean ± SEM. (**e**) Constitutively active SREBP2 (*N*-terminal; nSREBP2) promoted activity of sterol regulatory element (SRE) that regulates expression of cholesterol biosynthesis genes in HEK293T cells. (**f**) Expression of cholesterol biosynthesis genes in MDA-MB-231HM cells with constitutively active nSREBP2 and with or without exogeneous BMP4 expression. n = 3/group, mean ± SEM. Statistical analysis was completed using Student’s *t* test. ns, not significant; *, *p* < 0.05; **, *p* < 0.01.

As expected, enforced expression of BMP4 in 231-HM primary tumours led to a reduction in the levels of total and free cholesterol (Figure 4a). In 231-HM cells cultured in low serum-containing medium, enforced expression of BMP4 led to a significant reduction in expression of the cholesterol biosynthesis gene 3-hydroxy-3-methylglutaryl-CoA synthase 1 *(HMGCS1)* and a trend for reduced expression of 3-hydroxy-3-methylglutaryl-CoA reductase (*HMGCR*) and transmembrane 7 superfamily member 2 (*TM7SF2*) (Figure 4b).

The lack of profound suppression of cholesterol biosynthesis genes by BMP4 in vitro compared to that observed in primary tumours could be due to differences in the basal expression of these genes (Figure 4c). We noted that the transcript levels of *HMGCR*, *HMGCS1* and *TM7SF2* were significantly higher in 231-HM cells recovered from primary tumours compared to levels in cultured cells (Figure 4d).

As approximately 70% of genes in the SREBF (sterol regulatory element-binding protein, also abbreviated to SREBP) pathway were suppressed by BMP4 (Figure 3a), we next investigated the role of this pathway in BMP4-mediated suppression of cholesterol biosynthesis. SREBP1 and SREBP2 are known transcriptional regulators of cholesterol biosynthesis genes [34]. Given that BMP4 did not profoundly modulate the expression of SREBP1 or SREBP2 at the transcript level in vivo (1.13-fold and 0.74-fold, respectively, according to our RNA sequencing data [5]), we hypothesised that BMP4 might suppress the activity of SREBPs via a post-transcriptional mechanism. We therefore generated a lentiviral construct encoding an *N*-terminal constitutively active form of SREBP2 (nSREBP2) [34] and investigated if it could reverse BMP4-mediated suppression of cholesterol biosynthesis.

Using HEK293T cells, a cell line routinely used for the transient transfection of reporter constructs, we confirmed that enforced expression of nSREBP2 was effective in inducing a sterol regulatory element (SRE) that controls the expression of numerous cholesterol-related genes [35,36] in vitro (Figure 4e). However, this reporter assay could not be completed in MDA-MB-231HM cells due to their pre-existing expression of luciferase. Enforced expression of nSREBP2 in MDA-MB-231HM cells was not able to overcome BMP4-mediated suppression of cholesterol biosynthesis genes in vitro (Figure 4f). In addition, there was no difference in tumour growth or the extent of metastasis due to enforced expression of nSREBP2 in 231-HM-BMP4 tumours (Figure S1).

To investigate alternative pathways wherein SREBPs could be regulated post-translationally, we utilised the Cytoscape software 3.9.0 [37] and the BioGrid human protein–protein interaction database [38] and identified BMP4-regulated genes that are known to interact with SREBP2 at the protein level (Figure S2). Notable targets include ID2 and ID3 that antagonise SREBP1-mediated transcriptional regulation [39] and LINC00839 that interacts with SREBP2 in an E3 ubiquitin ligase complex [40].

### 2.4. Inhibition of Cholesterol Biosynthesis Suppresses Metastasis

To confirm the clinical relevance of BMP4-mediated suppression of cholesterol biosynthesis, we examined the expression levels and prognostic values of individual genes that are involved in cholesterol biosynthesis in the Metabric dataset [41]. The average expression of cholesterol biosynthesis-related genes (in C2-curated gene sets) was inversely correlated with the average expression of BMP4 and SMAD4, an indicator of canonical BMP4 signalling (Figure S3a). For the majority of these genes, expression was elevated in high-grade breast tumours and predicted worse overall survival of patients (Figure S3b–g).

**Figure 5:**
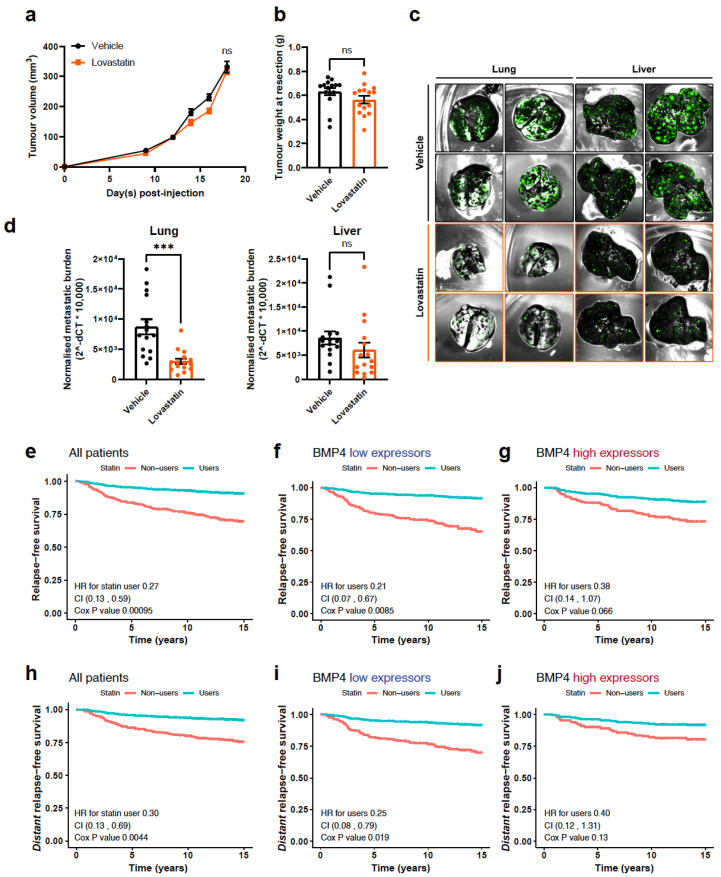
Statin therapy suppresses the metastasis of 231-HM xenograft tumours and is associated with a lower risk of breast cancer relapse in patients. (**a**) The effect of lovastatin on the growth of primary 231-HM tumours. 231-HM cells (1 × 106) were injected into the mammary glands of NSG mice. The mice were treated with lovastatin (10 mg/kg; 5 days/week) once the tumours became palpable following injection. n = 15/group, mean ± SEM. (**b**) The weights of resected 231-HM tumours. The tumours were resected on day 18 after tumour cell inoculation, when the average tumour volume reached approximately 400 mm3. n = 15/group, mean ± SEM. (**c**) Representative images of TurboGFP-tagged metastatic lesions in the lungs and livers visualised ex vivo using the Maestro imaging system. The mice were euthanised 15 days after tumour resection. (**d**) The normalised metastatic burden in the lungs (**left**) and livers (**right**) at the endpoint. n = 15/group, mean ± SEM. (**e**–**j**) The prognostic value of statin use in a cohort of 407 breast cancer patients. Relapse-free survival (**e**) and distant relapse free survival (**h**) in all patients in the cohort. (**f**,**i**): patients whose tumours were low for BMP4. (**g**,**j**): patients whose tumours were high for BMP4. Statistical analysis was completed using the survival: 3.1-11 package in R. ns, not significant; ***, *p* < 0.001.

The most commonly available therapies to lower cholesterol levels in patients are statins, and their potential impact on cancer progression is being investigated in multiple cancer types [42]. High cholesterol has been implicated previously as a factor in breast cancer progression and statins have been associated with primary and secondary protection [43]. We did not assess whether the migration of 231HM cells +/− BMP4 is altered by statin treatment in vitro, since previously we have reported that BMP4 does not alter the migration of breast cancer cells [4]. Instead, we tested the effect of a lipophilic statin, lovastatin, on the growth and metastasis of 231-HM tumours. Mice were treated daily (5 days per week) with lovastatin, commencing when the tumours were first palpable. Similar to the observations from BMP4-expressing tumours, lovastatin treatment did not alter the growth of primary tumours (Figure 5a). The tumours were resected on the same day, and no significant difference in the weights of tumours was found (Figure 5b). At the endpoint, significantly fewer metastases were observed in the lungs of mice that were treated with lovastatin, with a trend towards reduced liver metastasis as well (Figure 5c,d), partially replicating the anti-metastatic effect of BMP4. Our findings indicate that therapeutic inhibition of cholesterol biosynthesis may be a viable approach to suppress breast cancer metastasis, possibly with greater benefit for patients with low-BMP4-expressing tumours as exemplified by the 231-HM tumour line.

### 2.5. The Relationship between BMP4 and Statin Use in Breast Cancer Patients

We examined the effect of statin usage on breast cancer relapse in a cohort of 407 breast cancer patients (Table S1) for which we had previously revealed the prevalence of BMP4 at the protein level [4]. Patients who took statins had a significantly lower overall risk of relapse (Figure 5e) and of distant relapse (Figure 5h) from breast cancer. The benefit of statin use was most evident in patients whose tumours expressed low levels of BMP4 (Figure 5f,i), with a non-significant trend towards benefit in patients with high-BMP4 tumours (Figure 5g,j) (HRs for distant relapse-free survival, 0.25 and *p* = 0.019; 0.40 and *p* = 0.13, respectively). Considering metastatic sites, high levels of BMP4 correlated with a reduction in visceral metastases compared to low levels (4.3 vs. 13.3%, *p* = 0.017) but had no impact on bone metastases (9.8 vs. 9.9%, *p* = 0.96). Looking at statins separately, no significant site difference was seen, although usage was associated with a modestly greater numerical reduction in visceral metastases (6.9 vs. 10.6%, *p* = 0.206) than in bone metastases (8.5 vs. 7.6%, *p* = 0.766).

## 3. Discussion

In this study, we have re-analysed the transcriptomic data recovered from human cancer cells grown as xenografts in mice to characterise the impact of BMP4. Tumours derived from the triple-negative MDA-MB-231HM line, with or without exogenous BMP4 expression, were recovered and subjected to RNA sequencing in our previous study [5]. One of the genes downregulated by BMP4 was HMGCS1, a key enzyme in the cholesterol synthesis pathway [44]. Further analysis of the phosphoproteome in response to BMP4 revealed other genes involved in the regulation of cholesterol, including cell cycle and apoptosis regulator 2 (CCAR2), cohesion loading factor (NIPBL), NCL (nucleolin) and polybromo 1 (PBRM1). Gene set enrichment analysis confirmed that cholesterol biosynthesis-related pathways were strongly downregulated by BMP4. Consistent with earlier studies [45,46], we found that a commonly used statin, lovastatin, suppressed spontaneous metastasis to the lungs in the triple-negative MDA-MB-231HM tumours that had negligible BMP4 expression. In a cohort of breast cancer patients for whom outcome and therapy data were available, patients whose tumours had low levels of BMP4 protein demonstrated a reduced rate of relapse if on statin therapy.

Our observation that the master regulator of cholesterol synthesis, SREBP2, does not appear to be a major factor in the regulation of tumour growth and metastasis is consistent with another study demonstrating that RORγ, a member of the RAR-related orphan receptor family, can also act as the major driver of cholesterol biosynthesis in triple-negative breast cancer [31].

While biosynthesis of cholesterol is essential for cell viability and proliferation [47], its contribution to tumour growth and metastasis remains controversial [48]. The level of cholesterol dictates the fluidity of the cell membrane, with reduced cholesterol leading to increased membrane fluidity which is associated with epithelial-to-mesenchyme transition (EMT) and enhanced migration and invasion [49]. This group demonstrated that enhancing the level of free cholesterol in tumour cells decreased their membrane fluidity and decreased their migratory capacity. Further, agents that decreased membrane fluidity resulted in increased cholesterol in 4T1 cells and primary tumours, without altering primary tumour growth. However, spontaneous lung metastasis was reduced in treated mice [49]. In our study, we found that statin therapy also does not alter primary tumour growth, but in a setting with reduced systemic cholesterol, we found an inhibition of metastasis. However, we cannot directly ascribe this inhibition of metastasis to the reduced cholesterol synthesis within the tumour cells.

The observation that changes in membrane cholesterol levels do not alter primary tumour growth in our study, nor in Zhao et al. [49], at least following inoculation of a bolus of tumour cells into mice, indicates that cholesterol and/or membrane fluidity are not impacting tumour cell proliferation or tumour growth in this setting, but rather impacting the steps involved in subsequent metastasis.

Our results are more in line with the findings of Tang et al. [50] who reported that the enhancement of cholesterol synthesis in 4T1 and MDA-MB-231 tumour cells through knockdown of the negative regulator of SREBP2, ASPP2, led to elevated cholesterol levels, increased migration and invasion, and more lung metastases in a lung colonisation assay. Further, the knockdown of HMG-CoA reductase, the key enzyme in the cholesterol synthesis pathway, caused the opposite response of reduced cholesterol and a reduced ability to colonise the lung.

The widespread use of statins that inhibit HMG-CoA reductase has been very effective in controlling hypercholesterolemia and subsequent cardiovascular disease. Many studies have sought a link between statin use and either cancer prevention or improved prognosis, with the majority indicating no benefit for risk reduction [51]. However, for breast cancer, a meta-analysis of 17 studies concluded that statin use was associated with a lower risk of recurrence [43]. This is consistent with our data, where we found that relapse rates were reduced significantly in patients receiving statins.

In summary, we have identified cholesterol biosynthesis as a major process inhibited by BMP4 and confirmed that statin usage reduces cancer recurrence or metastasis in patients whose breast tumours have a low expression of BMP4. Thus, low BMP4 protein levels may be a predictive biomarker for additional therapeutic targeting of the cholesterol biosynthesis pathway.

## 4. Materials and Methods

### 4.1. Cell Culture and In Vitro Assays

MDA-MB-231HM (231-HM) cells were gifted by Prof. ZM Shao at the Fudan University Cancer Institute (Shanghai, China) and were authenticated by short tandem repeat (STR) profiling. HEK293T cells were obtained from ATCC. The cells were cultured in Dulbecco’s modified eagle medium (DMEM, Thermo Fisher Scientific, Waltham, MA, USA, #11965126), supplemented with 10% foetal calf serum (FCS, Bovogen, Keilor East, Australia, #SFBS) and penicillin/streptomycin (ThermoFisher #15140122), and maintained in a humidified incubator at 37 °C with 5% CO_2_. The cells were routinely screened using MycoAlert (Lonza, Basel, Switzerland, #LT07) and were negative for mycoplasma contamination.

### 4.2. Western Blotting

Western blotting analyses were completed as previously described [5]. Briefly, cells or tumours were mechanically homogenised and lysed in 2% SDS (50 mM Tris-HCl, pH 7) supplemented with 1 × Halt phosphatase/protease inhibitor cocktail (ThermoFisher Scientific #78428). The homogenate was incubated at 95 °C for 10 min and passed through a 26-gauge needle and syringe several times. Fifteen to fifty micrograms of proteins in loading buffer (0.1 M DTT, 4% glycerol and 0.0004% bromophenol blue in lysis buffer) were loaded onto Bolt Bis-Tris mini protein gels (ThermoFisher Scientific #NW0412C) and run at 200 V in MOPS buffer (ThermoFisher Scientific #NP0001) for approximately 35 min. The proteins were transferred onto PVDF membranes using a Bolt mini blot module at 20 V for 75 min. Membranes were blocked with 5% skim milk in PBS for 1 h and incubated with primary antibodies (ITGB4, 1:1000, Cell Signaling Technology, Danvers, MA, USA, #14803; ENPP1, 1:1000, Santa Cruz, Dallas, TX, USA, #sc-393419; HMGCS1, 1:1000 Santa Cruz #sc-166763; or HSP90, 1:5000, Abcam, Cambridge, UK, #ab203126) at 4 °C overnight or at room temperature for 2 h. Following incubation with HRP-conjugated secondary antibodies (BioRad, Hercules, CA, USA, #170-6515 or #170-6516) at room temperature for 1 h, protein bands were detected with the Western Lightning-Plus enhanced chemiluminescence (ECL) substrate (PerkinElmer, Waltham, MA, USA, #NEL103001EA).

### 4.3. Bioinformatics and Patient Dataset Analyses

RNA sequencing analyses were completed as previously described [5]. The dataset can be accessed at GSE199628. Briefly, differential gene expression analysis was completed using the edgeR: 3.28.1 [52] and limma 3.42.2 [53] packages. Further data analysis and visualisation was completed using the ComplexHeatmap 2.16.0 [54], ggplot2: 3.3.0 and ggpubr: 0.2.5 packages. Gene set enrichment analysis was completed and visualised using the clusterProfiler 4.8.3 and enrichplot 1.20.3 packages [55]. Analysis of the interactions between BMP4-regulated genes and SREBP1 or SREBP2 was completed using the Cytoscape 3.9.0 software [37] and the BioGrid human protein–protein interaction database [38].

Breast cancer patient datasets were either retrieved from the Metabric cohort on cBioPortal, which contains matched mRNA expression profiles and clinical information of 1904 patients [56], or from a breast cancer relapse cohort, as we reported previously (Table S1) [4]. Survival analysis was completed using the survival package.

### 4.4. Mass Spectrometric Analysis

Cell pellets were lysed in 4% sodium deoxycholate (SDC) at 95 °C for 5 min. A total of 500 µg of protein lysate was reduced and alkylated with 10 mM TCEP and 40 mM 2-chloroacetamide (CAM) at pH 7–8 at 95 °C for 5 min. Protein digest was completed using LysC and Trypsin at an enzyme-to-substrate ratio of 1:100 and incubated overnight at 37 °C with shaking at 1500 rpm.

For total proteomics, 50 μg of the digest protein was desalted through C18 columns and eluted in 50%ACN/0.1% TFA for mass spectrometry analysis. The remaining protein digest was subjected to global phosphoproteomic enrichment using TiO_2_, as previously described [57]. In brief, the removal of the SDC was performed through the addition of 400 µL of isopropanol (ISO) and 100 µL of 48% TFA/8 mM KH_2_PO_4_ solution. The samples were centrifuged at 2000× *g* for 10 min and the supernatant was collected. TiO_2_ beads (5 mg) were resuspended in 6% TFA/80% acetonitrile (ACN) (*vol*/*vol*) prior to being added to each sample. The samples were then incubated at 40 °C with shaking at 2000 rpm for 5 min. The TiO_2_ beads were then washed four times with 1 ml 5% TFA/60% ISO (*vol*/*vol*) and loaded onto C8 StageTips, and the phosphopeptides were eluted with 60 µL 5% ammonia solution/40% ACN (*vol*/*vol*). The eluates were dried in a vacuum concentrator (Labconco, Kansas City, MO, USA) to a volume of ~15 µL, resuspended in 1% TFA in ISO (*vol*/*vol*), transferred onto SDB-RPS StageTips and centrifuged to dryness at 1500 g. The SDB-RPS StageTips were subsequently washed with 1% TFA in ISO (*vol*/*vol*) followed by 0.2% TFA/5% ACN (*vol*/*vol*). The phosphopeptides were eluted with 0.1% ammonia solution/60% ACN (*vol*/*vol*) and dried down in a vacuum concentrator, prior to mass spectrometry analysis (Q-Exactive Plus Hybrid Quadrupole-Orbitrap, Thermo Scientific) at the Monash Proteomics & Metabolomics Platform (MPMP). 

The mass spectrometry raw files were processed using MaxQuant software (v6.12.0) with the following parameters: FDR < 0.01, precursor mass tolerance set to 20 ppm, fragment mass tolerance set to 0.5Da, a minimum peptide length of six amino acids, enzyme specificity set to Trypsin/P and LysC, human uniport database (v2020) and a maximum number of missed cleavages of 2. Fixed modifications were limited to Carbamidomethyl (C) and variable modifications were set to Oxidation (M), Acetyl (protein N-term), and Phospho (STY). The ‘match between runs’ option in MaxQuant was selected using the default parameters.

### 4.5. Cholesterol Quantitation

Cholesterol was extracted from resected primary tumours as described previously [31]. In brief, the tumours were homogenised mechanically in chloroform/isopropanol/Triton X-100 (7:11:0.1). The suspension was centrifuged to retain the organic phase that contained lipids and cholesterol, which was then dried in a vacuum concentrator and resuspended in isopropanol. Free and total cholesterol levels were quantitated using the Amplex red cholesterol assay kit (Thermo Fisher Scientific #A12216) and normalised to tumour weights.

### 4.6. Quantitative Reverse-Transcription PCR (RT-qPCR)

qPCR analyses were completed as previously described [5]. Briefly, total RNA from the cells was isolated using Trizol (ThermoFisher Scientific #15596026). Residual DNA was removed using the TURBO DNA-free kit (ThermoFisher Scientific #AM1907). cDNA was synthesised using the ProtoScript II reverse transcriptase (New England Biolabs, Ipswich, MA, USA, #M0368) with random pentadecamers. Gene expression was determined using the SYBR green real-time PCR master mix (ThermoFisher Scientific #4385612) with target-specific forward and reverse primers. Ribosomal protein L37a (*RPL37a*) was used as an internal control. For primer sequences, see Table S2.

### 4.7. Plasmid Cloning and Promoter Activity Assay

The sequences of the cloning primers used in this study can be found in Table S2. An *N*-terminal SREBP2 sequence (167–1624 of NM.004599.4, followed by an added stop codon) was cloned into the pLV-hygro backbone (Addgene, Watertown, MA, USA, #85134) [58]. Lentiviral particles were produced in HEK293T cells and used to infect 231-HM cells for the constitutive expression of nSREBP2.

An artificial promoter sequence consisting of a CCAAT box (CB1) from the *HMGCS1* gene, three consecutive repeats of sterol binding elements (SREs) and binding sites for a transcription factor Sp1, and a TATA box (attgg CAACTGGGCTCTCGT atcaccccac CCCGCC atcaccccac CCCGCC atcaccccac CCCGCC tata AGATCT) [35,36], was cloned into a modified pGL4.28 vector [59] between the *Nhe*I and the *Bgl*II restriction sites. Following stable lentiviral transduction of nSREBP2, HEK293T cells were transfected with the artificial promoter construct and incubated for 24 h. Promoter activity was measured using a luciferase assay system (Promega, Madison, WI, USA, #4030) according to the manufacturer’s instructions.

### 4.8. Mouse Experiments

All animal experiments were approved by the Austin Health Animal Ethics Committee prior to commencement. Animals were housed in a clean and temperature-regulated facility with free access to food and water. Tumour growth and metastasis experiments were completed as previously described [5]. Briefly, 1,000,000 turboGFP- and luciferase-tagged 231-HM cells in 1:1 PBS/Matrigel were injected into the fourth mammary fat pad of NOD scid gamma (NSG) mice. For some experiments, the mice were treated with lovastatin (10 mg/kg/day, 5 days/week via intraperitoneal injections) or a vehicle control. Tumour volume was monitored by calliper measurements and calculated as ½(length × width^2^). Tumours were surgically resected when they reached 400 mm^3^ in volume. The mice were humanely euthanised 15 days after surgery. Metastatic lesions in different organs were visualised ex vivo using the Maestro2 multispectral imaging system (CRi).

To quantitate metastatic burden at the endpoint, livers and lungs containing metastatic lesions were homogenised mechanically in lysis buffer (0.1 mg/mL proteinase K, 100 mM NaCl, 25 mM EDTA, 5% SDS and 10 mM Tris-HCl, pH 8) and digested at 55 °C overnight. Excess protein was precipitated through incubation with 0.7 volumes of saturated NaCl solution (>5 M) on ice for 30 min. Genomic DNA in the supernatant was extracted by using phenol/chloroform/isoamyl alcohol (25:24:1, supplemented with 0.1% 8-hydroxyquinoline) followed by ethanol precipitation. Metastatic burden was assessed by qPCR quantitation of tumour cell-specific TurboGFP DNA, with Rps27a as the quantity control in extracted genomic DNA and calculated as 2 ^ (CT_TurboGFP_ − CT_Rps27a_) × 10,000. The primers and probes used in this assay can be found in Table S2.

## Data Availability

The RNA sequencing data that were used as the basis of this study have been deposited at the NCBI Gene Expression Omni-bus (GSE199628) and are openly available [NCBI Gene Expression Omni-bus] [https://www.ncbi.nlm.nih.gov/geo/query/acc.cgi?acc=GSE199628 (last update May 2024)] [GSE199628].

## Supplementary Materials

The following supporting information can be downloaded at https://www.mdpi.com/article/10.3390/ijms25179160/s1.
