# Supplementary material for: BMP4-Induced Suppression of Breast Cancer Metastasis Is Associated with Inhibition of Cholesterol Biosynthesis
Source: Int J Mol Sci. 2024 Aug 23;25(17):9160. doi: 10.3390/ijms25179160 (PMC11395556; doi:10.3390/ijms25179160)
Supplement: Supplementary file 1 [file ijms-25-09160-s001.zip › ijms-3101090-supplementary.pdf]

Supplementary Figure S1

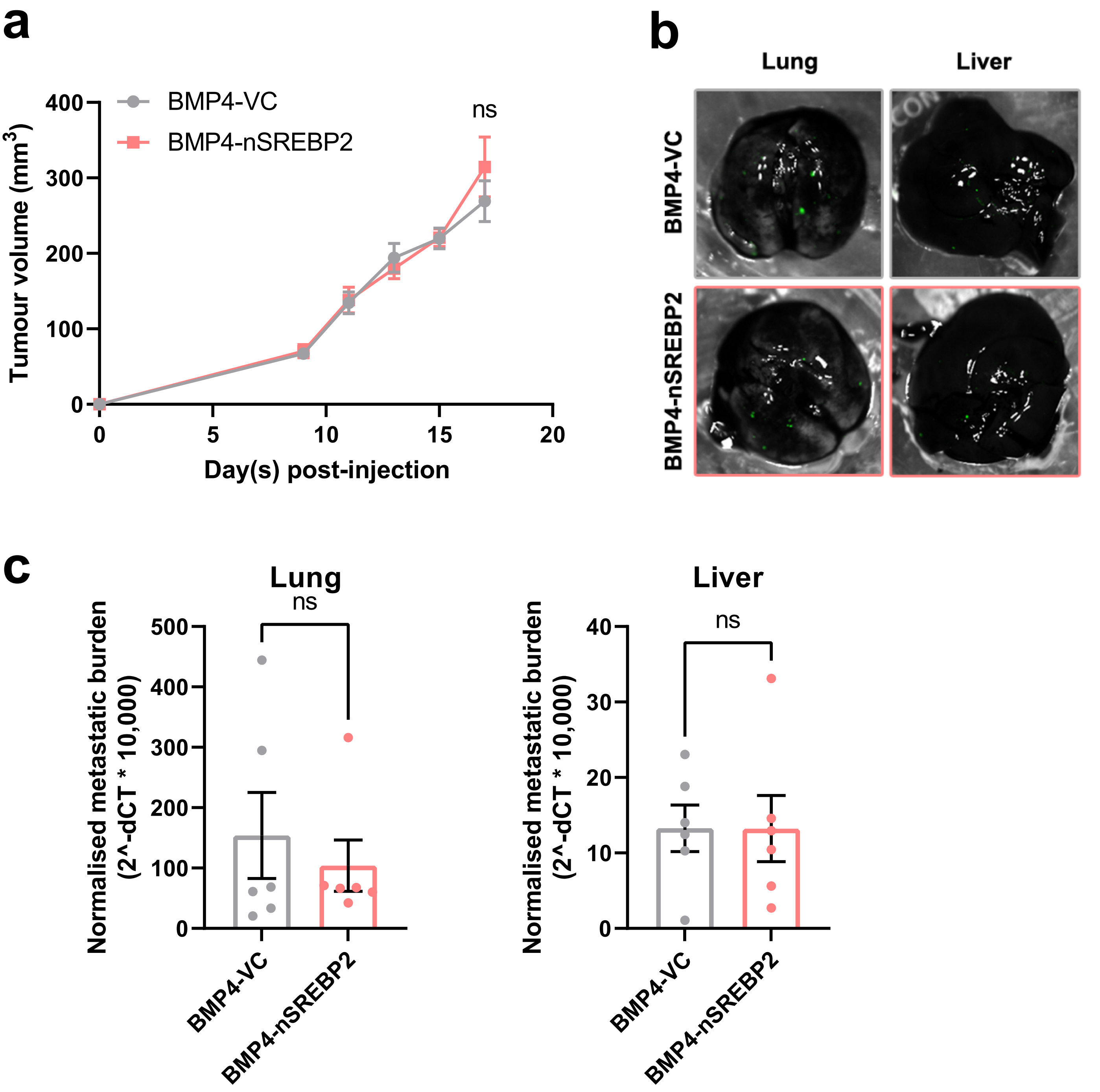

Supplementary Figure S2

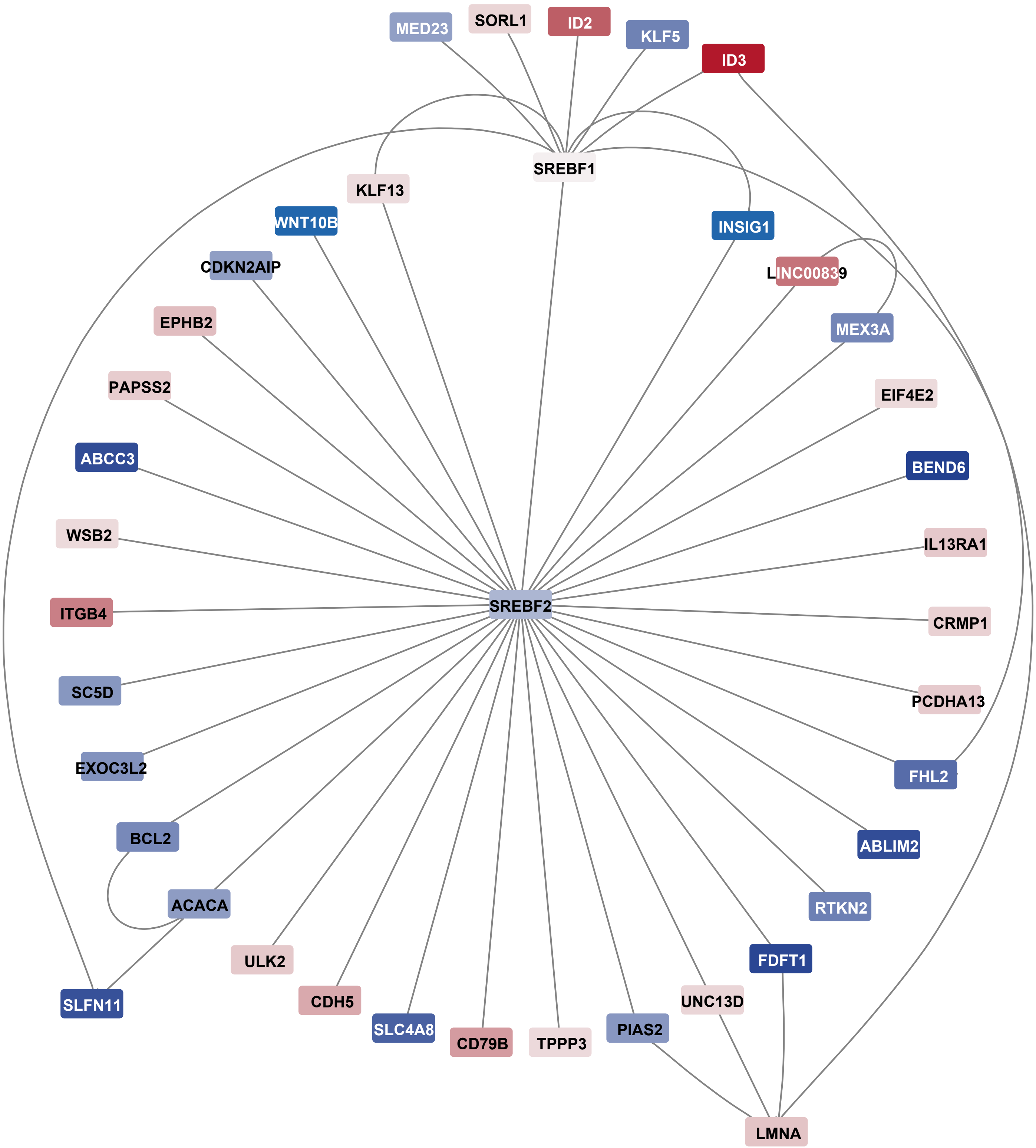

Supplementary Figure S3

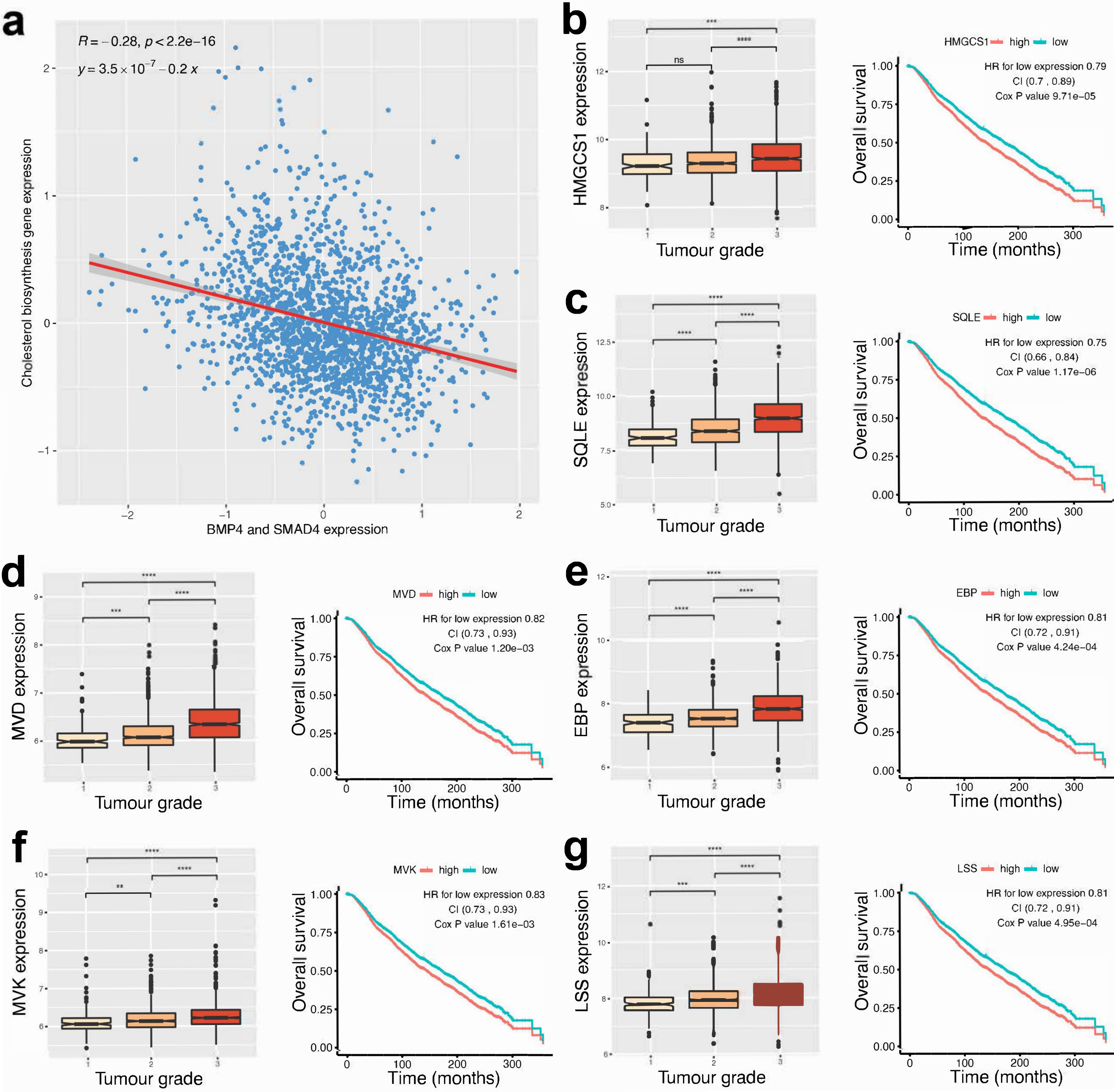

Figure S1. Expression of constitutively active nSREBP2 does not reverse BMP4-induced suppression of metastasis.

(**a**) Effect of enforced expression of constitutively active nSREBP2 on the growth of 231-HM primary tumours. Cells ($1x10^6$) were injected into the mammary glands of NSG mice. n=6/group, mean ± SEM.

(**b**) Representative images of TurboGFP-tagged metastatic lesions in the lungs and livers visualised *ex vivo* using the Maestro imaging system. Mice were culled 15 days after tumour resection.

(**c**) Normalised metastatic burden in the lungs (left) and livers (right) at endpoint. n=6/group, mean ± SEM.

Statistical analysis in a, b and d was completed using Student's t test. ns, not significant; *, p<0.05; **, p<0.01; ***, p<0.001; ****, p<0.0001.

Figure S2. Visualisation of BMP4-targeted genes (identified by RNA sequencing analysis) that have been reported to interact with SREBP1 and/or SREBP2. Interaction map generated using the Cytoscape software and the BioGrid human protein-protein interaction database. Red, upregulated by BMP4; blue, downregulated by BMP4.

Figure S3. Association of cholesterol biosynthesis genes with progression of human breast cancer. Data derived from the Metabric dataset.

(**a**) The average expression of cholesterol biosynthesis genes correlates negatively with the average expression of BMP4 and SMAD4. Statistical analysis completed using the ggplot2 and ggpubr packages in R.

(**b-g**) Higher expression of different genes involved in cholesterol biosynthesis correlates with a higher tumour grade and worse outcome in breast cancer patients. For analysis of gene expression in tumours of different grades, statistical analysis was completed using the ggplot2 and ggpubr packages in R. ns, not significant; *, p<0.05; **, p<0.01; ***, p<0.001; ****, p<0.0001. For survival analysis, patients were divided into high- or low-expression groups based on median gene expression. Statistical analysis was completed using the survival package in R.

Table S1. Demographics of breast cancer patients analysed in Figure S5.

Table S2. Primers and probes used in this study.

Table S1. Demographics of breast cancer patients analysed in Figure S5.

		BMP4				
		no		yes		
		n	%	n	%	p diff Chi Sq
**total**		215		192		
**median age**		60.2		59.8		
**age range**		28 to 91		31 to 89		
**Statin useage**	**No**	176	81.9	155	80.7	0.869
	**Yes**	39	18.1	38	19.3	
**Tumour size**	**median (mm)**	20		18		
	**T1**	107	49.7	111	57.8	0.101
	**T2**	98	45.6	71	37	
	**T3**	9	4.2	8	4.2	
	**T4**	1	0.4	2	1	
**LN status**	**negative**	143	66.5	137	71.4	0.292
	**positive**	72	33.5	55	28.6	
**histol type**	**IDC**	183	85.1	137	68.5	0.025
	**ILC**	24	11.1	36	20.4	
	**tubular**	8	3	17	8.9	
	**other**	0	0	2	1	
**Grade**	**1**	40	18.6	56	29.2	0.064
	**2**	112	52.1	96	50	
	**3**	63	29.3	40	20.8	
**ER and PR**	**ER neg/PR neg**	40	18.6	27	14.1	-
	**ER pos/PR neg**	21	9.8	20	10.4	
	**ER neg/PR pos**	7	3.3	5	2.6	
	**ER pos/PR pos**	123	57.2	106	55.2	
	**unk**	24	-	34	-	
**HR positive**	**no**	40	18.7	27	14.1	0.21
	**yes**	174	81.3	165	85.9	
	**unknown**	1	-	0	-	
**HER2 positive**	**no**	167	86.5	148	93.7	0.034
	**yes**	26	13.5	10	6.3	
	**unknown**	22	-	34	-	
**sub-type**	**Lum A**	117	58.5	120	69.4	0.084
	**Lum B**	44	22	27	15.6	
	**HER2-enrich**	5	2.5	3	1.7	
	**TNBC**	34	17	23	13.3	
	**unknown**	15	-	19	-	
	**Lum A**	117	58.5	120	69.4	0.03
	**non-Lum A**	83	41.5	53	30.6	
	**unknown**	15	-	19	-	

Table S2. Primers and probes used in this study.

**1. qPCR primers**			
**Primers / probes**	**Gene**	**Primer Sequence**	
hRpl37a_QF1	*Rpl37a*	GCCAGCACGCCAAGTACAC	
hRpl37a_QR1		CCCCACAGCTCGTCTCTTCA	
hHmgcr_QF1	*Hmgcr*	GTCCAGGTCAGGGGATGCCA	
hHmgcr_QR1		ACGGCTAGAATCTGCATTTCAGGGA	
hHmgcs1_QF2	*Hmgcs1*	GCTCGGCGTCCCACTCCAAA	
hHmgcs1_QR2		GCTGTGGCAGGGAGTCTTGG	
hTm7sf2_QF1	*Tm7sf2*	GAACTGCGACCCGGCCTCATC	
hTm7sf2_QR1		CAGAGGGCATCACCCACGTAGA	
**2. Cloning primers**			
**Primer/shRNA**	**Gene**	**Sequence**	**Restriction site**
MluI-Kozak-Srebp2-f3	*nSREBP2*	GCCGCAACGCGTGCCACCATGGACGACAGCGG	MluI + Kozak
HpaI-Stop-hSrebp2-r2		TGCTCCGTTAACTCAGACACACAGAAGAATCCG	HpaI
SREsp1-f	SRE reporter	GCAGCTAGCATTGGCAACTGGGCTCTCGTATCACCCCACCCCGCC	NheI
		ATCACCCCACCCCGCCATCACCCCACCCCGCCTATAAAGATCTGG	
SREsp1-r		CCAGATCTTTATAGGCGGGGTGGGGTGATGGCGGGGTGGGGTGAT	BglII
		GGCGGGGTGGGGTGATACGAGAGCCCAGTTGCCAATGCTAGCTGC	
**3. Metastatic burden assay primers**			
**Primers / probes**	**Gene**	**Primer Sequence**	
mRps27a_f1a	*Rps27a*	ATCACGCTCGAGGTACGGGC	
mRps27a_r1a		CAGAAACCCGCCCTCAAACACT	
mRps27a_p1a		TGCCGTTGAGGAAGCCAAGGCCTGACTGAGGCCC	
TurboGFP_f3	*TurboGFP*	CAGCGGCTACGAGAACCCCT	
TurboGFP_r3		GGCCTCGTAGCGGTAGCTGA	
TurboGFP_p3		AGGACGGCGGCGTGCTGCACGTGAGCT	
